# International Medical Congress

**Published:** 1885-07

**Authors:** 


### International Medical Congress.

At the recent meeting of the American Medical Association, held at New Orleans, action was taken which resulted in the re-organization and enlargement of the committee having in charge, the preliminary arrangements for the Congress in 1887. By that action, the committee was made to consist of a larger number of persons than before. This committee, within a few days, met at Chicago and revised the action of the former committee, and made a re-adjustment of various matters. The number of Sections of the Congress were reduced from nineteen to sixteen. The Section on Oral and Dental Surgery was omitted, but the subject was added to the Section on Surgery; it may be as efficient here, as in a separate Section, this doubtless was the opinion of the General Committee. It may be a question however, whether it would not work more efficiently as a Sub-Section, or a Division of the Section of Surgery. It would seem that the work pertaining to General Surgery and that of Oral and Dental Surgery conducted together, would be more than could be profitably accomplished. It is gratifying that Oral and Dental Surgery still has a recognition and a place in the work of the Congress. If the Dentists of the Country will show themselves to be able to stand side by side with the Surgeons, and sustain the position acceptably, it will be a result most gratifying to every true lover of our profession. An opportunity is here afforded, that would not have been given, had Oral Surgery been a separate Section as at first arranged. Of the Sections retained, only of four, were the Chairman changed, namely, Section 1, Medical Education, Legislation and Registration, Chairman, Dr. S. E. Chaille, of New Orleans. Section 9, Ophthalmology, Chairman, Dr. E. Williams, of Cincinnati. Section 13, Laryngology, Chairman, J. W. McKenzie, of Baltimore. Section 18, Diseases of Children, Chairman, Dr. J. Lewis Smith, of New York. The remaining twelve Sections were left unchanged. If the way should continue to be open, as would how seem, we see no reason why the Dentists of the country, so far as they may be qualified for membership, may not enter heartily into their appropriate work in the Congress. It is to be hoped that no antagonism or embarrassment will arise, to lessen the good work that might be thus accomplished.

